# Design and Implementation of a Multi-Mode Telemetry Transmitter

**DOI:** 10.3390/s25175565

**Published:** 2025-09-06

**Authors:** Francesco Silino, Fabio Dell’Acqua, Anna Vizziello, Diego Biz, Francesco Costa, Pietro Savazzi

**Affiliations:** 1 Department of Electrical, Computer and Biomedical Engineering, University of Pavia, 27100 Pavia, Italy; francesco.silino01@universitadipavia.it (F.S.); fabio.dellacqua@unipv.it (F.D.); anna.vizziello@unipv.it (A.V.); 2 Temis S.R.L., 20011 Corbetta, Italy; diego.biz@temissrl.com (D.B.); francesco.costa@temissrl.com (F.C.)

**Keywords:** telemetry transmitter, continuous phase modulation, re-configurable hardware, Mean Squared Error (MSE)

## Abstract

In space applications, the required levels of performance and reliability drive up hardware costs. Reducing the efforts related to device development and validation may help balance the budget. A versatile transmitter for space telemetry is implemented here that may help in this respect. Such a device can switch across different linear and continuous phase modulation schemes just by modifying its parameters, while maintaining the same hardware structure. Results from an extensive campaign of experimental test measurements of the device are reported. A GNURadio-implemented receiver is developed to test performance of the actual transmitter by considering all the main blocks of the receiver chain and computing the bit error rate (BER) at the receiver. After testing different configurations, results confirm that the BER of the improved one-filter modulated signal is lower than the BER obtained using only the first Laurent decomposition component.

## 1. Introduction

Space communications enable data transmission between satellites and ground stations. Within this domain, Pulse Code Modulation/Frequency Modulation (PCM/FM) and Staggered Offset Quadrature Phase Shift Keying (SO-QPSK) are among the most commonly used modulation techniques. These methods are characterized by relatively low data rates and high robustness, which make them suitable for the long-distance links involved in space communication [1]. However, they exhibit limitations in terms of spectral efficiency, particularly PCM/FM, which offers low data throughput relative to its bandwidth consumption. Consequently, the need is growing for novel techniques aimed at enhancing both hardware flexibility and the performance of transmitters employing these modulation schemes.

Hardware flexibility can be reached by seamlessly switching among linear modulation architectures with higher spectral efficiency, leveraging the possibility of using the Laurent Decomposition of PCM/FM and SO-QPSK modulations to use equivalent linear models for modulation processes. This technique decomposes the continuous phase modulation (CPM) signal into a superposition of pulse amplitude modulated (PAM) components, effectively moving up the system nonlinearity from after the filtering stage to before it. By summing the filtered decomposition components, the original signal can be reconstructed [2].

In [3], a transmitter was designed to reduce the hardware complexity of the linearized PCM/FM modulation by using an approximation of the exact Laurent decomposition based on a single-filter implementation. The single-filter implementation was based on minimizing the mean square error (MSE) between the approximated modulation and its exact representation.

While Laurent decomposition is typically applied at the receiver side for simplified signal approximation, the study in [3] adopts an alternative, transmitter-side approach. This enables dynamic switching between different modulation schemes with minimal configuration changes. Furthermore, the Laurent decomposition may also be used for modulation index estimation [4] or channel variation analysis [5]. Hence, the transmitter [3] can seamlessly switch from PCM/FM to SO-QPSK by applying a variant of the Laurent decomposition known as Perrins and Rice decomposition [6].

A central challenge with Laurent decomposition lies in the exponential growth in the number of required filters when the pulse length L is extended. It is well established, however, that a substantial portion of the signal energy is concentrated in the output of the first filter—exceeding 90% for PCM/FM—and within the first two filters for SO-QPSK and similar schemes. This is the reason why in [3] the filter was optimized using a MSE minimization technique, thereby enhancing performance without increasing the number of coefficients or requiring more than one filter.

**Figure 1:**
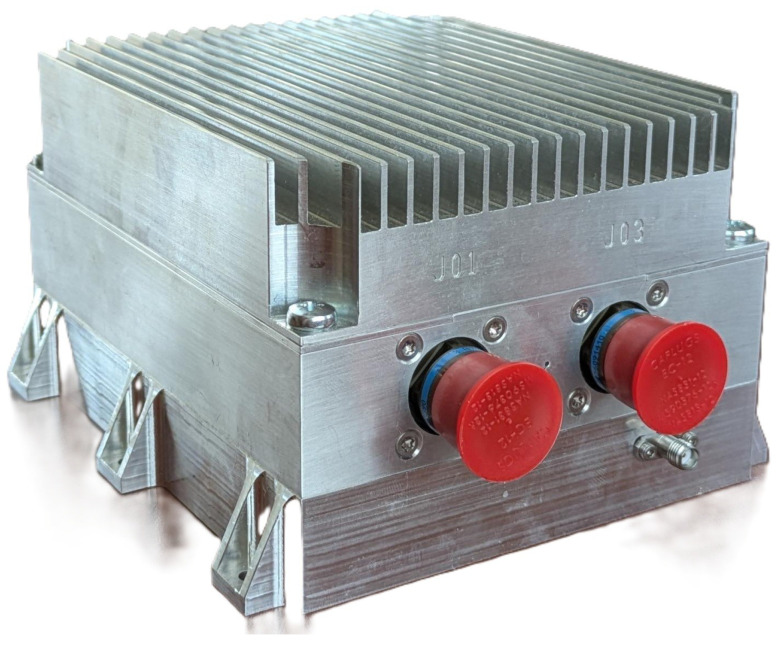
TLM-TX box.

In this work, the scheme proposed in [3] is implemented and experimentally validated via the hardware implementation of the entire PCM/FM transceiver. Figure 1 shows the custom-built transmitter prototype. The full transceiver has been tested using a software-defined radio (SDR) receiver implemented in a radio frequency (RF) evaluation board, with GNURadio [7] used for both the transmit and receive sides.

Owing to its linear filtering structure driven by pulse-shaped symbols, the architecture inherently supports the generation of widely used digital modulation formats such as M-ary quadrature amplitude modulation (M-QAM), without requiring modifications to the transmitter hardware. This flexibility enables potential performance optimization under varying environmental conditions.

The main contribution of this paper is the practical realization and experimental validation of the multi-mode telemetry transmitter architecture previously proposed only at a conceptual or simulation level. Specifically, this work implements the proposed Laurent decomposition-based linear modulation on real FPGA hardware, integrating it with a software-defined radio receiver for comprehensive testing. This advancement represents a fundamental step towards the development of a flexible and reconfigurable telemetry transmitter suitable for space applications, bridging the gap between theoretical design and deployable technology.

The remainder of this paper is organized as follows. Section 2 introduces the PCM/FM modulation and the implemented linear approximation based on one single filter. Section 3 describes the hardware employed for the telemetry transmitter implementation. Section 4 presents the receiver architecture developed using GNURadio. Section 5 details the transmission test results. Finally, Section 6 concludes the paper and outlines directions for future works.

## 2. PCM/FM Modulation

### 2.1. CPM Signal

A CPM signal can be represented by

(1) $$s\left(t,\alpha \right)=e^{j\psi (t,\alpha )}$$

where

(2) $$\psi \left(t,\alpha \right)=2h\pi \sum_{i}\alpha_{i}q\left(t,iT\right)$$

where *h* denotes the modulation index, *T* represents the signaling interval, $\alpha_{i}$ stands for the information symbol, and *q*(*t*) is the phase response of the system. The relationship between q(t) and the frequency response *f*(*t*) is expressed by the following equation:

(3) $$q\left(t\right)=\int_{-\infty }^{t}f(\tau )d\tau$$

The pulse f(t) depicted in (3) is constrained in time to (0,LT), where *L* represents the phase pulse duration in terms of symbol intervals, and it must adhere to the following conditions:

(4) f(t)=f(LT−t)

(5) $$\int_{0}^{LT}f(\tau )d\tau =q\left(LT\right)=\frac{1}{2}$$

In the subsequent section, we will outline the standard method for achieving the nonlinear signal presented in Equation (1) via a linear modulation technique.

### 2.2. Laurent Decomposition

As demonstrated in [8], the right-hand side of Equation (1) for binary modulation can be represented as a combination of PAM waveforms. This methodology was further developed for *M*-ary symbols in [2].

(6) $$s\left(t,\alpha \right)=\sum_{k=0}^{Q-1}\sum_{n}b_{k,n}c_{k}\left(t-nT\right)$$

where $Q=2^{L-1}$ and $c_{k}\left(t\right)$ is expressed as

(7) $$c_{k}\left(t\right)=\prod_{i=0}^{L-1}u(t+iT+\beta_{k,i}LT),\;\;\;0\leq k\leq Q-1$$

The function u(t) is defined as

(8) $$u\left(t\right)=\left\{\begin{array}{cc} \sin[2h\pi q(t)]/\sin(h\pi ), & 0\leq t\leq LT\\ \sin[h\pi -2h\pi q(t)]/\sin(h\pi ), & LT\leq t\leq 2LT\\ 0, & \mathrm{elsewhere} \end{array}\right.$$

The parameter $\beta_{k,i}$ is either 0 or 1, except $\beta_{k,0}$, which is always zero. For any *i* such that 1≤i≤L−1, $\beta_{k,i}$ corresponds to the *i*-th bit in the binary representation of *k* as follows:

(9) $$k=\sum_{i=1}^{L-1}2^{i-1}\beta_{k,i}\;\;\;\;\;\;0\leq k\leq Q-1$$

Pseudosymbols, denoted as the symbols at the input of the linear filters $c_{k}\left(t\right)$ in (6), specifically $b_{k,n}$, are calculated from the true input symbols $\alpha_{i}$, as described in Equation (10):

(10) $$b_{k,n}=exp\left\{jh\pi \left[\sum_{m=-\infty }^{n}\alpha_{m}-\sum_{i=0}^{L-1}\alpha_{n-i}\beta_{k,i}\right]\right\}$$

## 3. PCM/FM Transmitter Implementation

### 3.1. Main Transmitter Specifications

The telemetry transmitter, in accordance with standard space communication practices, operates within the S-band spectrum allocated for satellite communications. Specifically, its design supports a configurable center frequency within the 2.2–2.3 GHz range. The RF output power is fixed at 10 W and tuned during production. The system achieves a maximum spectral bandwidth of 20 MHz, which is derived from a modulation rate of 40 mega samples per second (Msps), i.e., useful sample rate, which is further interpolated prior to digital-to-analog conversion and subsequent quadrature upconversion.

As the development was driven by customer-defined requirements, the link-budget analysis and other associated calculations (e.g., bandwidth, modulation scheme, output power, data rate, antenna gain, and path losses) were performed during the initial design phase and are therefore not included here. These parameters are selected to ensure a robust communication link from ground stations to at least Low Earth Orbit (LEO) and Medium Earth Orbit (MEO).

The remaining components of the telemetry system are not fully specified, as only the intermediate frequency (IF) description with its relevant datasheet were required for development. It should be noted that the telemetry transmitter accepts a synchronous serial bitstream, with accompanying clock, as input, along with power supply and control signals for enabling the RF high-power amplifier. It provides feedback regarding internal temperature and the operational status of the high-power RF amplification stage. The input/output interfaces are deliberately simplified to enhance system reliability and minimize configuration errors.

### 3.2. Radio Frequency Design

**Figure 2:**
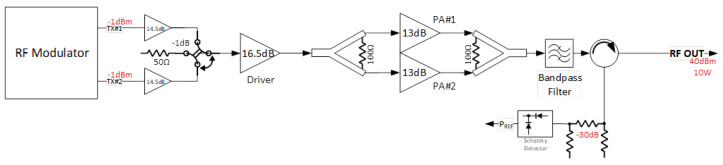
Radio frequency scheme.

To enhance resilience against radiation-induced events, the radio frequency (RF) modulator architecture employs a dual-modulator configuration, as shown in Figure 2. This redundant design enables the generation of identical RF signals through two independent modulation chains, each feeding a dedicated output. Each modulator output is connected to a pair of low-power amplifiers, followed by an RF switch. The switch selects the path delivering the appropriate RF output power, thereby mitigating the impact of Single Event Effects (SEEs), such as temporary or permanent failure of one modulation chain. The selected RF signal is then routed to a Gallium Nitride (GaN) driver stage, which provides the necessary gain for high-power amplification.

The driver output is split to feed two high-power GaN field-effect transistors (FETs), whose outputs are subsequently combined and filtered to produce the final transmission signal. An RF circulator is employed to protect the system from antenna mismatch conditions. The circulator’s reverse port is monitored for reflected power, enabling the system to disable the high-power amplifier in the presence of excessive Voltage Standing Wave Ratio (VSWR). The GaN FET technology was selected due to its inherent robustness and radiation tolerance, making it well suited for space-based applications.

### 3.3. Baseband Signal Description

The main parameters of the transmitter modulation were configured as follows:

- L=2;
- h=0.7;
- $\alpha_{i}=\pm 1$;
- T=1;
- $T_{c}=1/8$ (sampling time).

where $T_{c}$ represents the sampling time in the digital baseband, and all other symbols were defined in the previous section.

A 6th-order Bessel filter is needed for PCM/FM, but a Raised Cosine (RC) filter serves as a suitable approximation and is more practical for digital implementations. The impulse response defining the RC filter, which approximates the 6th-order Bessel filter, is given by

(11) $$f\left(t\right)=\left\{\begin{array}{cc} \frac{1}{2LT}\left[1-cos(\frac{2\pi t}{LT})\right], & 0\leq t\leq LT\\ 0, & \mathrm{otherwise} \end{array}\right.$$

The effectiveness of the RC filter is on par with, or potentially exceeds, that of a Bessel filter [9].

To construct the complex envelope of the PCM-FM modulated signal using Equation (6), the filter impulse response $c_{k}\left(t\right)$ is determined for L=2. This computation relies on the parameters set earlier and involves applying Equations (7) and (8).

The pseudosymbols $b_{k,n}$ are determined through the use of Equation (10), specifically when L=2, as

(12) $$\beta_{k,i}=\left(\begin{array}{cc} 0 & 0\\ 0 & 1 \end{array}\right)$$

#### One-Component Approximation with Enhancement Performance

To enhance the performance achievable with a single linear component representation, a suitable filter defined as

(13) $$c_{w}=\left[c_{w}\left(0\right),c_{w}\left(1\right),\ldots ,c_{w}\left(N-1\right)\right]$$

was obtained by solving the subsequent Wiener–Hopf equation [10] defined as

(14) $$c_{w}=R_{b_{0}}^{-1}r_{s\left(t,\alpha \right)b_{0,n}}$$

Here, $R_{b_{0}}$ represents the autocorrelation matrix for the pseudosymbol sequence $\left\{b_{0,n}\right\}$, while $r_{s\left(t,\alpha \right)b_{0,n}}$ signifies the correlation between the modulated signal $s(nT_{c},\alpha )$ and the pseudosymbols $\left\{b_{0,n}\right\}$. The values for these autocorrelation and cross-correlation matrices have been determined by averaging across more than $8\times 10^{5}$ samples. Equation (14) minimizes the mean square error (MSE) between the comprehensive modulated signal representation and the signal derived by filtering the pseudosymbols $\left\{b_{0,n}\right\}$ from (6) using $c_{w}$. A lower bound for the achievable mean square error is determined by the following formula [10]:

(15) $$\epsilon =r_{b_{0}}\left(0\right)-r_{s\left(t,\alpha \right)b_{0,n}}^{H}R_{b_{0}}^{-1}r_{s\left(t,\alpha \right)b_{0,n}}$$

where $r_{b_{0}}\left(0\right)$ is the autocorrelation of the pseudosymbols $\left\{b_{0,n}\right\}$ computed in 0, i.e., their variance, while the other quantities are defined as in (14).

Using $c_{w}$ instead of the pair $c_{0}+c_{1}$ allows us to reduce the complexity for L=2 by removing one of the filters while still achieving better performance than using only $c_{0}$. For L>2, the number of required filters in the Laurent representation increases, resulting in even greater hardware reduction when using $c_{w}$. According to the definition of the number of filters $Q=2^{L-1}$ given after Equation (6), for L=3, the number of required filters rises to four, whereas the proposed architecture still employs only one.

### 3.4. FPGA Performance Evaluation

The FPGA employed in the telemetry transmitter is a commercial off-the-shelf (COTS) component not specifically designed for space applications. Hence, it was subjected to radiation testing to characterize its sensitivity and identify the types of radiation-induced effects it may experience. Based on the results of these tests, the firmware development and place-and-route phases were meticulously adapted to either mitigate or eliminate the impact of such effects. Mitigation strategies include the use of Triple Modular Redundancy (TMR) synthesis, filtering techniques, and reconfiguration mechanisms. This approach was deemed both practical and effective by the customer, the European Space Agency (ESA), particularly considering that the equipment operates outside the atmosphere for a limited timespan (on the order of a few hours). The relatively short exposure time, combined with the adopted development methodology, results in reliability and availability metrics that meet the specified mission requirements.

The practical objective of this work led to the implementation and testing of a field programmable gate array (FPGA) design using a hardware description language (HDL)-based simulation. This approach took into account various hardware implementation factors, including 16-bit fixed-point operations with signals quantized to 12 bits at the input of an AD9361 (Analog devices, Cambridge, MA, USA) digital-to-analog quadrature upconverter used in the final transmitter implementation.

**Figure 3:**
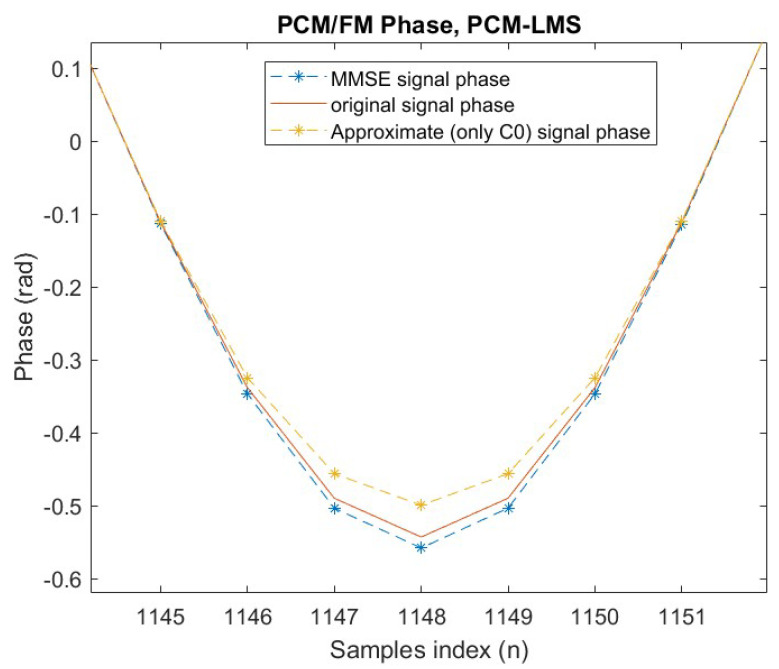
Phase comparison detail.

The FPGA operates at 40 Msps. Therefore, to align the results with MATLAB 2024a outputs at an oversampling rate of 8, the FPGA’s output data rate was configured to 5 Mbps. To assess the resemblance of the proposed single-filter modulated signal to the original nonlinear signal, Figure 3 illustrates a comparison among the MMSE signal phase, the phases obtained via the full Laurent decomposition, and the approximate reconstruction utilizing only the $c_{0}$ component. These phase trends are plotted across a set of samples chosen randomly from those deemed to have the most significant comparisons.

It can be seen that the MSE approximated phase aligns more closely with the full nonlinear phase compared to the Laurent approximation using only $c_{0}$.

**Figure 4:**
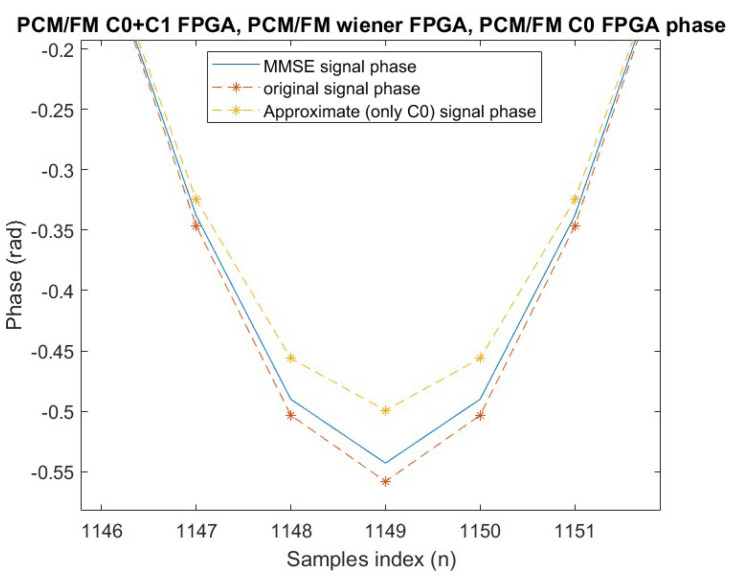
FPGA: phase comparison detail.

Figure 4 presents a similar comparison, now focusing on signals that have been reconstructed in the FPGA simulation implementation.

Table 1 presents a numerical comparison featuring the MSE values for various approximate representations. To maintain brevity, results are shown for the oversampled signal only. In the last row of the table, the lower bound for the MSE is also reported, which was computed as in (15).

The MMSE $c_{w}$ filter reported a 3.7 dB improvement in performance over utilizing merely the initial component, $c_{0}$, from the Laurent representation, while it was only 2.9 dB higher than the lower bound. The 0.1 dB discrepancy when compared to the floating-point results obtained with Matlab arises from the fixed-point representation employed in HDL simulations, which use 12 bits for signals and 16 bits for internal processes. Although these results were initially obtained through FPGA simulations, they were subsequently validated in real hardware implementations, as their accuracy inherently depends solely on the finite-precision arithmetic employed.

## 4. GNURadio Transceiver

**Figure 5:**
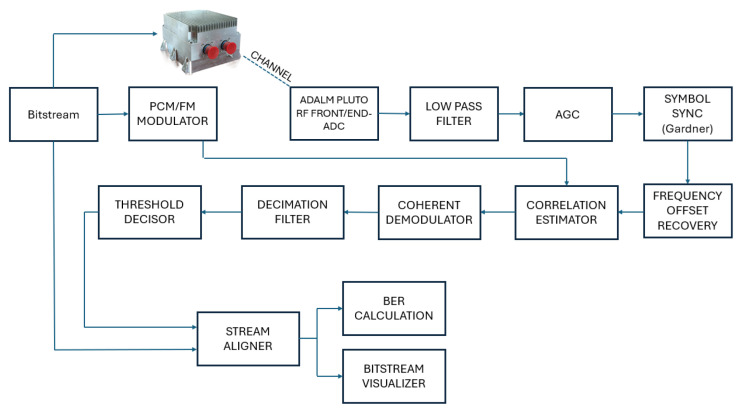
GnuRadio transceiver structure.

To evaluate the performance of the implemented transmitter in FPGA, a GNUradio-based [11] transceiver was developed. This transceiver was used to receive the signal transmitted by the telemetry transmitter developed in the FPGA. The telemetry transmitter implemented Laurent’s decomposition, using either the $c_{0}+c_{1}$ filter or the single-filter realization, with the possibility of selecting only the $c_{0}$ filter or the one optimized through Wiener filtering. The purpose of this testbed was to validate the innovative technique proposed in [3], as well as the TEMIS telemetry transmitter, in a real transmission environment. The system blocks shown in Figure 5 were implemented using the communication library blocks, adapting their parameters to meet the specific system requirements and parameters.

In the following, the transmitter and receiver systems are described. The GNURadio-based transceiver is detailed in [12].

### 4.1. Transmitter

The transmitter module in GNURadio was designed to generate a reference PCM/FM signal for comparison with the signal received from the telemetry transmitter. The same bit sequence is used for both the FPGA-based telemetry transmitter and the GNURadio-generated signal, ensuring accurate benchmarking.

The transmitted sequence consists of a Consultative Committee for Space Data Systems (CCSDS) standard preamble (4 bytes), followed by 1279 bytes of random data. This packet is repeated and modulated by both systems, allowing for the comparison of the spectrum and other characteristics of the modulated PCM/FM signal. Signal modulation in GNURadio is performed using the CPM Modulator block, which is configured according to standard PCM/FM parameters.

To ensure bit-level synchronization, the Tagged Stream Align block is used, enabling precise data tagging within GNURadio. This allows for synchronization between transmitted and received bitstreams, which is crucial for accurate bit error rate (BER) evaluation. The modulation parameters are consistent with those used in the MATLAB simulations.

### 4.2. Receiver

The receiver module performs signal acquisition using the ADALM-Pluto SDR and executes the necessary recovery steps, including symbol, frequency, and timing offset corrections.

Initially, the acquired signal is passed through a low-pass filter to remove out-of-band noise. Subsequently, an automatic gain control (AGC) mechanism normalizes the signal constellation to the unit radius. Symbol synchronization is performed using the Gardner algorithm [13], followed by frequency offset correction through an open-loop algorithm based on Machine Learning (ML) techniques [14].

Once synchronization is complete, a Correlation Estimator block computes the correlation between the received signal and the known preamble sequence. When the correlation exceeds a defined threshold, the system triggers frame alignment by tagging the start of the received frame. After synchronization, quadrature demodulation is applied, and the signal is passed through a decimation filter to complete the demodulation process.

## 5. Telemetry Transmitter Tests

As previously discussed, the telemetry transmitter employs Laurent decomposition, supporting both the $c_{0}$ and $c_{0}+c_{1}$ filter configurations. Additionally, it implements a custom approach that replaces $c_{0}$ with the novel filter $c_{w}$, representing the first decomposition component optimized by minimizing the MSE with the exact signal representation.

The measurement setup consists of the following components:

- A telemetry transmitter with dedicated power supply;
- An SMA coaxial cable connecting the transmitter’s Tx port to the receiver’s Rx port (ADALM-Pluto board);
- An ADALM-Pluto board connected via USB to a PC for signal acquisition and processing.

The communication was established at a frequency of 2.3 GHz. To improve test stability and achieve more precise results, a direct cable connection was used in place of an antenna, thereby eliminating environmental interferences. Nevertheless, a successful over-the-air transmission test was also performed using antennas to validate the system’s performance under typical operating conditions.

The transmission power was configured to 10 dBm. The transmitter sends a repeated CCSDS-compliant packet, enabling real-time observation of both the modulated signal and the corresponding received bitstream in GNURadio. The received data are aligned with the original transmitted sequence, allowing for accurate correlation analysis and performance evaluation.

**Figure 6:**
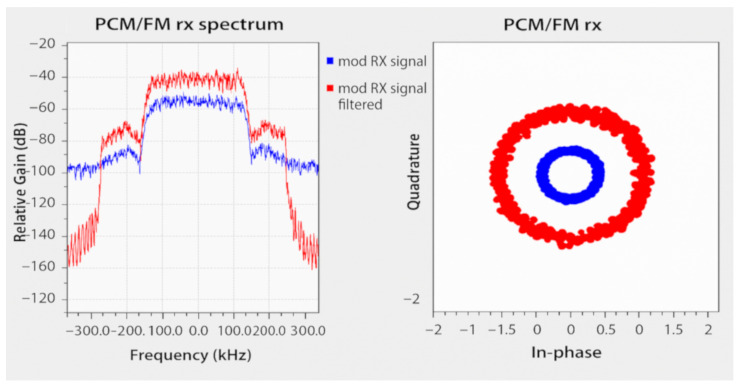
Spectrum, constellation, and BER of $c_{w}$ modulated signal.

Figure 6 shows the spectrum, constellation diagram, and BER of the signal transmitted by the telemetry transmitter (TLM-TX) using the $c_{w}$ filter in the modulation process.

### Results Analysis

The transmitted and received sequences were acquired and stored in the GNURadio binary format. For a comprehensive evaluation of the system performance, the data were subsequently imported into MATLAB for post-processing. Whereas in previous simulations only the MSE was assessed, in this round, the BER was also computed.

The MSE was first evaluated on the received signal. Prior to the computation, the signal power was normalized to 1 to reduce numerical inaccuracies. It is important to note that the received signal had already undergone AGC processing. Due to the inherent characteristics of CPM used in PCM/FM, the absolute values of the received symbols differ from those of the transmitted ones. This is expected, as in this modulation scheme the information is not encoded in the absolute phase but rather in the phase difference between successive symbols. Consequently, the MSE was computed based on the phase difference between two consecutive symbols, as expressed in the following:

(16) $$\mathrm{MSE}=\frac{\sum_{n=0}^{N-1}{\left|x_{o}\left(n\right)-x_{a}\left(n\right)\right|}^{2}}{N}$$

where *N* is the total number of samples, $x_{o}\left(n\right)$ represents the signal phase difference of the complete modulated signal, i.e., that uses both $c_{0}$ and $c_{1}$ filters, while $x_{a}\left(n\right)$ is the received signal phase difference obtained by using the MMSE filter $c_{w}$. As previously described, the phase difference samples $x_{o,a}\left(n\right)$ are computed as

(17) $$x_{o,a}\left(n\right)=\arg\left\{s_{o,a}\left(n-1\right)s_{o,a}^{*}\left(n\right)\right\}$$

where $s_{o,a}\left(n\right)$ is, respectively, the sampled complete modulated and received approximated signals at time instant *n*.

Table 2 presents the MSE values for both the simulated and received signals.

The results show that the MSE for both transmitted and received symbols improved when the $c_{w}$ filter was used. The SNR of the signal was calculated from the MSE, as explained in Equation (18).

(18) $$\mathrm{SNR}=10\log_{10}\left(\frac{sig_{pw}T_{c}}{mse}\right)$$

where $sig_{pw}$ is the normalized signal power, and mse is the linear mean square error.

The SNR and BER values for the different received signals, computed over $2.5\times 10^{6}$ symbols, are shown in Table 3. These BER results refer to raw, uncoded transmission; while the differences may appear marginal, even modest improvements at this level can lead to appreciable gains when channel coding is applied, as is typically done in real telemetry systems.

**Figure 7:**
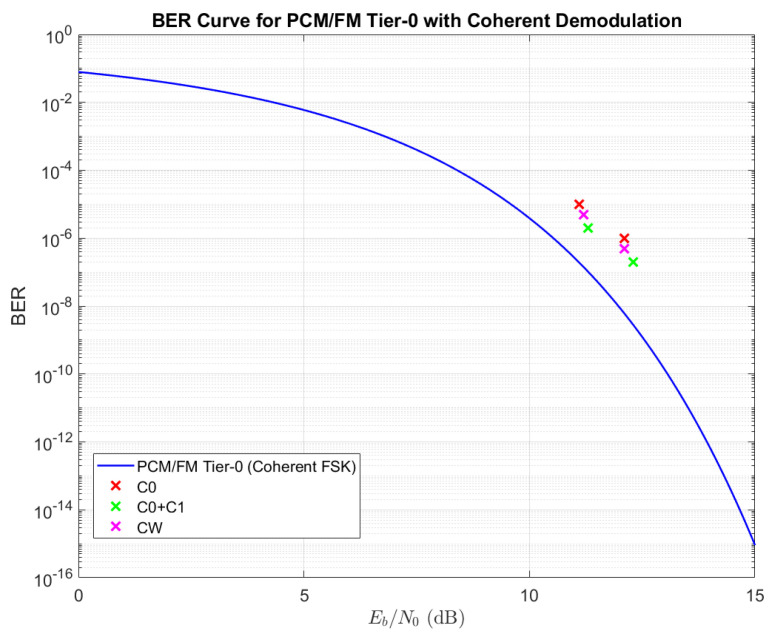
Comparison of experimental results with the PCM/FM BER curve.

To facilitate comparison, the BER curve for PCM/FM demodulated using a coherent demodulator is presented in Figure 7, where the points corresponding to experimental results are reported in the form of colored ‘×’ symbols. The cluster to the right was obtained through direct measurement; the cluster to the left was obtained by adding noise to the recorded experimental data and simulating the receiver performance in MATLAB. In both cases, the SNR value was computed *a posteriori*, which explains the slight vertical misalignment visible in each cluster.

It can be observed that the signal modulated using the $c_{w}$ filter achieved a better BER compared to the $c_{0}$ filter at the same signal-to-noise ratio (SNR). The points representing the received signals are displaced above the theoretical curve. This deviation is attributable to the non-ideal nature of the real signal reception and demodulation process, which introduces some losses. Consequently, this phenomenon results in a degradation of the BER for the demodulated signal.

As previously discussed, this paper focuses on the implementation of the developed transmitter on real hardware. In this context, the final transmitter prototype is presented in Figure 1.

## 6. Conclusions

This study presents the hardware implementation and experimental validation of an innovative linear architecture for space telemetry transmitters. The experimental setup demonstrates that the design offers two major benefits: a reduced number of hardware components required for signal reconstruction and enhanced versatility in supporting multiple modulation schemes on the same space-certified platform.

After conducting simulations and tests within the FPGA framework, the suggested solution exhibited a 3.7 dB enhancement in MSE for the modulated signal when compared to the first component of the Laurent decomposition. Remarkably, this improvement was accomplished without escalating the system complexity. Instead, complexity was minimized by employing a single signal-reconstructing filter on the transmitter side.

Within the realm of space communications and systems, where hardware and energy are limited and costly, an important benefit of this transmitter is its SDR-like characteristics. This feature enables the same space-certified platform to fulfill various roles by merely reprogramming it. Moreover, enhancements, including the incorporation of more advanced modulations like SO-QPSK and linear M-QAM, can be achieved via software updates.

The proposed method based on the minimization of the MSE through Wiener filtering can be easily applied to other similar nonlinear modulations. Hence, in future studies, we aim to apply the suggested decomposition method to the first two elements of the Perrin–Rice representation in the context of SO-QPSK modulation. Moreover, the performance obtained will be compared with other linearization techniques, and we plan to test the physical realization of the transmitter in environments with varying SNRs and data rates. A promising avenue for future advancement involves expanding the suggested transmitter into a distributed Multiple-Input, Multiple-Output (MIMO) framework, as outlined in [15].

## Figures and Tables

**Table 1:** FPGA MSE comparison.

Tx Filter	Oversampled Signal
$c_{0}+c_{1}$	−67.9 dB
$c_{0}$	−29.7 dB
$c_{w}$	−33.4 dB
$c_{w},\epsilon$	−36.3 dB

**Table 2:** MSE comparison between simulation and experimental implementation.

Tx Filter	Simulated Signal	Received Signal
$c_{0}+c_{1}$	−548.7 dB	−36.7 dB
$c_{0}$	−69.4 dB	−36.3 dB
$c_{w}$	−82.1 dB	−36.5 dB

**Table 3:** Signal SNR and BER.

Tx Filter	SNR	BER
$c_{0}+c_{1}$	12.3 dB	<4 $\times 10^{-7}$
$c_{0}$	12.1 dB	$3.6\times 10^{-6}$
$c_{w}$	12.1 dB	$8\times 10^{-7}$

## Data Availability

The original contributions presented in this study are included in the article. Further inquiries can be directed to the corresponding author.
